# Letter regarding “ *SCN9A* variant in a family of mixed breed dogs with congenital insensitivity to pain”—Navigating the pathogenicity of candidate gene mutations: Spotlight on paralog Nav genes

**DOI:** 10.1111/jvim.16708

**Published:** 2023-04-21

**Authors:** Taisuke Ishikawa, Hiroshi Aoki

**Affiliations:** ^1^ Hiranomachi Pet Clinic Nagasaki Japan

Dear Editors,

We read the recent publication by Dr. Gutierrez‐Quintana et al. titled “*SCN9A* variant in a family of mixed breed dogs with congenital insensitivity to pain” with great interest.^1^ While mutations in voltage‐gated sodium channel (Nav) genes, including *SCN9A*, are widely recognized in human medicine as a cause of inheritable neurological disorders, this paper published in JVIM is noteworthy not only for being the first report of canine *SCN9A* disease with clear genetic evidence, but also appears to be the first report of a sodium channelopathy in dogs. In their study, the authors conducted whole‐genome sequencing analysis of a dog with congenital insensitivity to pain and identified the pathogenic variant c.C2761T (p.R921C) in *SCN9A*, which encodes the alpha subunit of voltage‐gated neuronal sodium channel Nav1.7 that is mainly expressed in nociceptive neurons.^2^ Furthermore, the authors speculated that the Nav1.7‐R921C variant is disease‐causing based on five perspectives: evolutionarily conserved amino acid sequences, comparisons with a large amount of genetic data of healthy controls, in‐silico functional prediction, functional impacts of arginine substitution, and reference to human clinic data. Although these findings are sufficient to suggest that *SCN9A*‐C2761T is the causative mutation in this rare condition, they do not conclusively align with the official guideline of the American College of Medical Genetics and Genomics and the Association for Molecular Pathology (ACMG‐AMP) based on experience in genetic diseases of humans.^3^ In this letter to the editor, we propose that further functional evaluation of the genetic variant be conducted to strengthen the causal link to the disease.

We would like to discuss the authors' procedure for evaluating the genetic variant. First, the authors used the orthologous alignment of Nav1.7 amino acid sequences across multiple species to demonstrate that the Nav1.7‐R921C mutation is pathogenic. This method is a solid proof of concept based on the idea that mutations in evolutionarily conserved amino acid sequences of certain protein can cause significant functional changes. Second, the authors compared whole genome sequence data of the affected dog with those of 926 healthy control dogs, and found that the *SCN9A*‐C2761T allele was absent in controls. The fact that the candidate variation was isolated using large genomic data emphasizes the significance of *SCN9A*‐C2761T. Third, the authors used three in‐silico prediction programs, and determined Nav1.7‐R921C as deleterious. However, in‐silico tools generally have low specificity and tend to over‐predict missense mutations as pathogenic. Therefore, the ACMG‐AMP guideline recognizes that in‐silico prediction is a weak evidence annotation tool to assign pathogenicity.^3^ Fourth, the authors speculated that Nav1.7‐R921C was a functionally altered variant by explaining the substitution of a positively charged arginine residue with cysteine. This “arginine‐substitution” theory is likely true if the substitution occurs in the transmembrane domain S4, which is rich in arginine residues to function as a voltage sensor to mediate channel opening.^4^ However, because Nav1.7‐R921C is expected to locate on the pore‐forming loop between transmembrane domains S5 and S6 on Uniprot,^5^ another functional explanation might be given to this substitution. Fifth, there is a case record of the homologous genetic variation Nav1.7‐R922C in a human with hereditary sensory and autonomic neuropathy (accession number; VCV000836792) in the ClinVar database.^6^ However, even if a similar record is found in ClinVar, which has collected millions of human genetic variants from clinical settings, the identification of single record does not necessarily mean that the genetic variant is more likely to cause disease, if it is a single record. It is important to note that the current rating for human Nav1.7‐R922C in ClinVar is “uncertain significance.”

Taken together, the authors' evaluation of the variant provides some evidence for its pathogenicity, but may not be sufficient to conclusively determine its causality. To minimize false‐positive results, the ACMG‐AMP guideline in human genetics recommends a comprehensive evaluation of various information including family segregation, literature review, functional analysis of the mutated protein, and frequency data in the general population. However, in veterinary clinics, it is often challenging to obtain such data due to limited genomic databases, difficulties in addressing siblings, and the scarcity of similar publications for rare diseases. To bridge the gap between ensuring disease causality and the limitations of genetics in veterinary clinics, we propose that functional annotations of mutated proteins, such as enzymatic assays in metabolic diseases or electrophysiological assays in arrhythmic diseases, could be a valuable tool in determining pathogenicity for some inherited disorders.

Fortunately, we can use an established electrophysiological technique called patch clamp to measure the function of genetic variants in ion channel genes, including *SCN9A*, by recording ion current through the cell membrane and better understanding ion channel behavior. Although there are no reports of functional studies on the canine Nav1.7‐R921C or the human Nav1.7‐R922C variants, studying the functional effects of mutations in paralogous Nav families could be useful. Brugada syndrome (BrS) is a lethal arrhythmia in humans that is inherited and associated with mutations in *SCN5A*, which encodes the alpha subunit of Nav1.5, a channel highly expressed in the heart.^7^ A human Nav1.5‐R893C variant, homologous to the canine Nav1.7‐R921C variant, has been repeatedly reported as pathogenic in BrS,^8^ in contrast to only one record of human Nav1.7‐R922C in ClinVar. Brugada syndrome is characterized by a loss of sodium channel function, similar to Nav1.7 mutations in congenital insensitivity to pain. To our surprise, our whole‐cell patch clamp experiment showed that the Nav1.5‐R893C variant had markedly decreased sodium currents,^7^ suggesting that the Nav1.7‐R921C variant would also markedly decrease sodium currents. We are confident that this experimentally‐acquired functional annotation of the human Nav1.5‐R893C variant could provide further evidence for the malignant pathogenicity of the canine Nav1.7‐R921C variant.

In summary, some convincing evidence has been presented to support the pathogenicity of *SCN9A*‐C2761T, resulting in Nav1.7‐R921C. Additionally, we here demonstrated further evidence of loss of sodium channel function of Nav1.7‐R921C, based on quantitative experiments with its homolog, human Nav1.5‐R893C. The authors have clearly demonstrated a link between an *SCN9A* mutation and congenital pain insensitivity, and we believe it would be useful to sequence *SCN9A* in dogs with similar congenital pain insensitivity and perform functional analysis of gene variants in veterinary practice. If the functional annotation is challenging, we suggest following the paralog mutations and supplementing the data with references to functional analysis data in similar human diseases, such as sodium channelopathies caused by mutations in *SCN5A*.

## CONFLICT OF INTEREST DECLARATION

Authors declare no conflict of interest with regard to this letter.
